# Effects of 4′-Demethylnobiletin and 4′-Demethyltangeretin on Osteoclast Differentiation In Vitro and in a Mouse Model of Estrogen-Deficient Bone Resorption

**DOI:** 10.3390/nu15061403

**Published:** 2023-03-14

**Authors:** Michiko Hirata, Tsukasa Tominari, Ryota Ichimaru, Naruhiko Takiguchi, Yuki Tanaka, Masaru Takatoya, Daichi Arai, Shosei Yoshinouchi, Chisato Miyaura, Chiho Matsumoto, Sihui Ma, Katsuhiko Suzuki, Florian M. W. Grundler, Masaki Inada

**Affiliations:** 1 Department of Biotechnology and Life Science, Tokyo University of Agriculture and Technology, 2-24-16 Nakacho, Koganei, Tokyo 184-8588, Japan; 2 Cooperative Major of Advanced Health Science, Tokyo University of Agriculture and Technology, 2-24-16 Nakacho, Koganei, Tokyo 184-8588, Japan; 3 Faculty of Sport Sciences, Waseda University, 2-579-15 Mikajima, Tokorozawa, Tokyo 359-1192, Japan; 4 Institute of Crop Science and Resource Conservation, University of Bonn, Karlrobert-Kreiten-Strasse 13, 53115 Bonn, Germany; 5 Life Science Inada Unit, Institute of Global Innovation Research, Tokyo University of Agriculture and Technology, 2-24-16 Nakacho, Koganei, Tokyo 184-8588, Japan

**Keywords:** 4′-demethylnobiletin, 4′-demethyltangeretin, osteoclast, postmenopausal osteoporosis

## Abstract

Citrus nobiletin (NOB) and tangeretin (TAN) show protective effects against disease-related bone destruction. We achieved demethylation of NOB and TAN into 4′-demethylnobiletin (4′-DN) and 4′-demethyltangeretin (4′-DT) using enzyme-manufacturing methods. In this study, we examined the effects of 4′-DN and 4′-DT on in vitro osteoclast differentiation, and on in vivo osteoporotic bone loss in ovariectomized (OVX) mice. 4′-DN and 4′-DT clearly suppressed the osteoclast differentiation induced by interleukin IL-1 or RANKL treatment. 4′-DN and 4′-DT treatments resulted in higher inhibitory activity in osteoclasts in comparison to NOB or TAN treatments. RANKL induced the increased expression of its marker genes and the degradation of IκBα in osteoclasts, while these were perfectly attenuated by the treatment with 4′-MIX: a mixture of 4′-DN and 4′-DT. In an in silico docking analysis, 4′-DN and 4′-DT directly bound to the ATP-binding pocket of IKKβ for functional inhibition. Finally, the intraperitoneal administration of 4′-MIX significantly protected against bone loss in OVX mice. In conclusion, 4′-DN, 4′-DT and 4′-MIX inhibited the differentiation and function of bone-resorbing osteoclasts via suppression of the NF-κB pathway. Novel 4′-DN, 4′-DT and 4′-MIX are candidates for maintaining bone health, which may be applied in the prevention of metabolic bone diseases, such as osteoporosis.

## 1. Introduction

Bone homeostasis is precisely regulated by the balance of osteoclastic bone resorption and osteoblastic bone formation. Interference in bone homeostasis is caused by various bone metabolic diseases, such as age-related osteoporosis in men and estrogen withdrawal-related osteoporosis in women. One of the key molecules of these metabolic bone diseases is the receptor activator of the NF-κB ligand (RANKL). RANKL expressed on the cell surface of osteoblasts is an essential molecule that participates in the differentiation and activation of osteoclasts. RANKL interacts with RANK expressed on osteoclast precursors, to induce osteoclast differentiation and bone resorption [1,2]. Pro-inflammatory molecules, including interleukins (ILs), tumor necrosis factor (TNF)-α and prostaglandins (PGs), induce the expression of RANKL in osteoblasts to promote bone resorption [3,4].

Osteoporosis is characterized by low bone mass, which results in an increased risk of fractures in elderly individuals in Japan. Estrogen deficiency in postmenopausal women causes severe systemic bone loss, which is also called postmenopausal osteoporosis. Ovariectomized (OVX) mice, prepared by surgical excision of the ovaries, are generally used as an experimental model of postmenopausal osteoporosis. Previous studies reported that treatment with bisphosphonates, selective estrogen receptor modulators, an anti-RANKL antibody, parathyroid hormone and an anti-sclerostin antibody prevented bone loss in postmenopausal osteoporosis [5,6,7].

On the other hand, the utilization of nutrimental supplements, such as polyphenols, is a current trend in bone health maintenance. Polymethoxyflavones (PMFs), a family of flavonoids in citrus, are well-known polyphenols. We previously reported that PMFs exhibited antiosteoclastic activities, suggesting that they may be an effective compound for the treatment of postmenopausal osteoporosis and inflammatory periodontal bone resorption. Nobiletin (3′,4′,5,6,7,8-hexamethoxyflavone (NOB)) suppressed IL-1-induced osteoclast differentiation via the inhibition of NF-κB signaling in osteoblasts and the intraperitoneal injection of NOB restored bone mass in OVX mice [8]. The local injection of NOB and tangeretin (4′,5,6,7,8-pentamethoxyflavone (TAN)) into gingival tissues suppressed LPS-induced alveolar bone loss in mice [9]. Heptamethoxyflavone (3,3′,4′,5,6,7,8-heptamethoxyflavone (HMF)) inhibited LPS-induced bone-resorbing activity in bone cultures of mouse alveolar bone [10]. The intraperitoneal injection of a PMF mixture, consisting of NOB, TAN, HMF and 4′,5,6,7-tetramethoxyflavone, significantly inhibited bone loss in OVX mice and the mixture suppressed bone-resorbing activity in organ cultures of mouse alveolar bone [11]. Ohyama et al. reported that sudachitin (5,7,4′-trihydroxy-6,8,3-trimethoxyflavone), found specifically in *Citrus sudachi*, blocked LPS-induced calvarial bone destruction by repressing the ERK and JNK pathways and decreasing intracellular reactive oxygen species (ROS) production in osteoclast precursors [12]. These reports have suggested that PMFs are potential candidates for maintaining bone health.

We achieved the demethylation of NOB and TAN using enzymatic manufacturing methods that finally produced 4′-demethylnobiletin (4′-DN) and 4′-demethyltangeretin (4′-DT). Several studies have reported the biological activities of 4′-DN and 4′-DT. Li et al. showed that the anti-inflammatory property of 4′-DN is higher than that of NOB [13,14,15]. Wu et al. reported that 4′-DN inhibited the NF-κB-dependent pro-inflammatory pathway, whereas it activated Nrf2 (nuclear factor-erythroid 2-related factor 2)-dependent anti-oxidative pathways [16]. Guo et al. reported that 4′-DT suppressed the LPS-induced inflammatory response in macrophages via the inhibition of the NF-κB-dependent pro-inflammatory pathway and 4′-DT exhibited more potent activity than TAN [14]; however, the effects of these demethylated compounds on bone resorption remain unclear.

In the present study, we examined the effects of 4′-DN, 4′-DT and 4′-MIX (a mixture of 4′-DN and 4′-DT) on osteoclast differentiation in vitro and on osteoporotic bone loss in ovariectomized (OVX) mice in vivo. We examined the effects of 4′-DN, 4′-DT and 4′-MIX on bone loss in a mouse model of postmenopausal osteoporosis.

## 2. Materials and Methods

### 2.1. Animals and Reagents

Newborn, 6-week-old male and 8-week-old female *ddY* mice were obtained from Japan SLC Inc. (Shizuoka, Japan). 8-week-old female mice were randomly divided into three groups: sham (n = 6), OVX (n = 6), and OVX + 4′-MIX (n = 7). All procedures were performed in accordance with the institutional guidelines for animal research from the committee at the Tokyo University of Agriculture and Technology (protocol number: R03-158). Recombinant human IL-1α was purchased from R&D Systems (Minneapolis, MN, USA). Recombinant human soluble RANK ligand (sRANKL) was obtained from PeproTech Inc. (Cranbury, NJ, USA). NOB, TAN, 4′-DN, 4′-DT and 4′-MIX (a mixture of 4′-DN and 4′-DT) were supplied by Fuji Sangyo (Kagawa, Japan). The preparation of large amounts of the 4′-MIXwas prepared from citrus peel with enzymatic processing and liquid chromatography purification, mixed at a ratio of 1.0:0.6 (4′-DN:4′-DT).

### 2.2. Osteoclast Differentiation in Cocultures of Mouse Primary Osteoblasts and Mouse Bone Marrow Cells

Mouse primary osteoblasts (POBs) were isolated from newborn mouse calvariae by five routine sequential digestions with an enzyme cocktail of 0.1% collagenase (Fujifilm Wako Pure Chemical Corp., Osaka, Japan) and 0.2% disperse (Roche Diagnostics K.K., Tokyo, Japan). Mouse bone marrow cells (BMCs) were collected from tibial bone marrow of 6-week-old mice. BMCs were cocultured with POBs with or without IL-1 (2 ng/mL) and each PMF for 7 days in αMEM (Thermo Fisher Scientific Inc., Waltham, MA, USA) supplemented with 10% fetal bovine serum (Nichirei Bioscience Inc., Tokyo, Japan) and penicillin-streptomycin (Thermo Fisher Scientific Inc.) and osteoclasts were stained to detect tartrate-resistant acid phosphatase (TRAP). TRAP-positive multinucleated osteoclasts were counted.

### 2.3. Osteoclast Differentiation in Cultures of Raw264.7 Cells

Raw264.7 cells, a murine macrophage cell line, were cultured in the presence of sRANKL (100 ng/mL) with or without each PMF for 4 days. Osteoclasts were stained for TRAP and TRAP-positive multinuclear cells with three or more nuclei per cell were counted.

### 2.4. TRAP Staining

TRAP buffer was prepared to mix 0.1 M sodium acetate (Fujifilm Wako Pure Chemical Corp.) and 0.1 M acetate (Fujifilm Wako Pure Chemical Corp.). Naphthol AS-Mix phosphate (Merck KGaA., Darmstadt, Germany) was diluted with *N*,*N*-Dimethylformamide (Fujifilm Wako Pure Chemical Corp.). Then, naphthol solution and fast red violet LB salt (Merck KGaA.) were diluted with TRAP buffer to prepare TRAP-staining solution. Cells were fixed with 10% formalin solution (Fujifilm Wako Pure Chemical Corp.) for >10 min. Fixed cells were stained with TRAP-staining solution for 20 min.

### 2.5. Analysis of mRNA Expression by Quantitative PCR

Raw264.7 cells were cultured in the presence of sRANKL (100 ng/mL) with or without each PMF for 4 days. Total RNA was extracted from Raw264.7 cells. Total RNA was quantified with NanoDrop Lite (Thermo Fisher Scientific Inc.). Reverse transcription was conducted on 5 μg of total RNA using a Superscript II preamplification system (Thermo Fisher Scientific Inc.) to prepare cDNA. Then, cDNA was amplified by quantitative PCR (qPCR) with primer pairs designed on Primer3Plus (Whitehead Institute for Biomedical Research, Cambridge, MA, USA). PCR primer pairs were obtained from Eurofins Scientific (Luxembourg, Luxembourg). The sequences of the mouse PCR primer pairs were as follows: *Actb* (β-actin): (forward) 5′-ccccattgaacatggcattg-3′ and (reverse) 5′-acgaccagaggcatacagg-3′; *Ctsk* (Cathepsin K): (forward) 5′-cattctcagacacacaatccac-3′ and (reverse) 5′-gatactggacaccactggga-3′. qPCR was performed with SsoAdvanced SYBR Green Supermix (BioRad Laboratories Inc., Hercules, CA, USA) using CFX Connect Real-Time PCR System (BioRad Laboratories Inc.). The relative normalized gene expression, determined using the ΔΔCq method, was quantified using Bio-Rad CFX Manager 3.1 (BioRad Laboratories Inc.) and β-actin was used for normalization.

### 2.6. Analysis of Protein Expression by Western Blotting

Raw264.7 cells were cultured in the presence of sRANKL (100 ng/mL) with or without each PMF for 15 min. Raw264.7 cells were lysed in a lysis buffer containing PhosSTOP (Roche) and complete protease inhibitor cocktail EASYPack (Roche). The whole cell lysates were centrifuged at 12,000× *g* for 10 min and the supernatant was collected. The protein concentration of the supernatant was measured using a bicinchoninic acid (BCA) protein assay kit (Thermo Fisher Scientific Inc.). The 10 μg of protein in each sample was applied to SDS-PAGE with 10% polyacrylamide gel and transferred onto polyvinylidene difluoride membranes (Merck KGaA.). Membranes were blocked with 5% dry milk in PBS-T (PBS with 0.05% Tween-20) and incubated with primary antibodies at 4 °C overnight. Membranes were incubated with the corresponding secondary antibody in 1% skim milk in PBS-T and developed with ECL prime Western blotting detection reagent (GE Healthcare Japan Corp.) by ChemiDoc XRS+ (Bio-Rad Laboratories Inc.). Primary antibodies against IκBα (35–41 kDa; Santa Cruz Biotechnology Inc.) and β-actin (43 kDa; Santa Cruz Biotechnology Inc.) were used.

### 2.7. In Silico Molecular Docking Simulation

The three-dimensional X-ray crystal structure inhibitor of NF-κB kinase (IKK) β protein was obtained from a protein databank (PDB ID:4kik) [17]. For docking simulations, default parameters (H-atoms) were added into the protein structures using AutoDock Tools version 1.5.6 (Molecular Graphics Laboratory, La Jolla, CA, USA). The chemical structures of 4′-DN and 4′-DT were optimized using the online compound editor InDraw (http://in.indraw.integle.com [accessed on 6 June 2022]; Integle Chemistry, Inc., Shanghai, China). All two-dimensional structures were converted into three-dimensional structures in the pdb format and saved in the mol format using Open Babel (http://www.openbabel.org/ [accessed on 6 June 2022]) [18]. The protein–ligand molecular docking study was performed using AutoDock Vina version 1.2.0 (Molecular Graphics Laboratory) [19]. Subsequently, AutoDock Vina was used to implement fast docking of the inhibitor ligand into the active pocket of both the IKKβ and kinase domains, which considered the flexibility and mobility of the ligand molecules and protein active site residues, and used the Lamarckian genetic algorithm to fully explore the conformational space for the IKKβ inhibitor interactions. The rotational bonds of the protein were kept rigid, while those of the ligands were treated as flexible. The amino acids Leu21, Gly22, Thr23, Val29, Ala42, Lys44, Glu61, Val74, Met96, Glu97, Tyr98, Cys99, Gly102, Asp103, Glu149, Asn150, Val152, Ile165, Asp166 and the surrounding residues within a distance of 6.5 Å were defined as active ATP-binding sites. Three-dimensional docking models were created using BIOVIA Discovery Studio Visualizer version 21.1.0.20298 (Dassault Systèmes, Vélizy-Villacoublay, France).

### 2.8. Intraperitoneal Administration of a Mixture of Demethylated Compounds to OVX Mice

Eight-week-old female mice were either sham-operated or ovariectomized (OVX). 4′-MIX (2 mg/mouse/day) was intraperitoneally administered to mice daily for 4 weeks. After 4 weeks, the femurs were collected and the bone microarchitectures were analyzed by micro-computed tomography (µCT) (R_mCT2; Rigaku Corp., Tokyo, Japan). The following bone architecture parameters were analyzed: bone volume/tissue volume (BV/TV (%)), bone mineral contents/tissue volume (BMC/TV (mg/cm^3^)), trabecular number (Tb.N (1/mm)), trabecular separation (Tb.Sp (µm)).

### 2.9. Statistical Analysis

All data were expressed as the median or mean ± standard deviation (SD) with individual data points. A one-way ANOVA followed by Tukey’s test, as a post hoc analysis, was used for comparisons among three or more groups. All statistical analyses were performed using the GraphPad Prism 9 software program version 9.5.1 (GraphPad Software, San Diego, CA, USA).

## 3. Results

### 3.1. Comparative Effects of NOB, TAN and Its Demethylated Compounds on Osteoclast Differentiation

**Figure 1:**
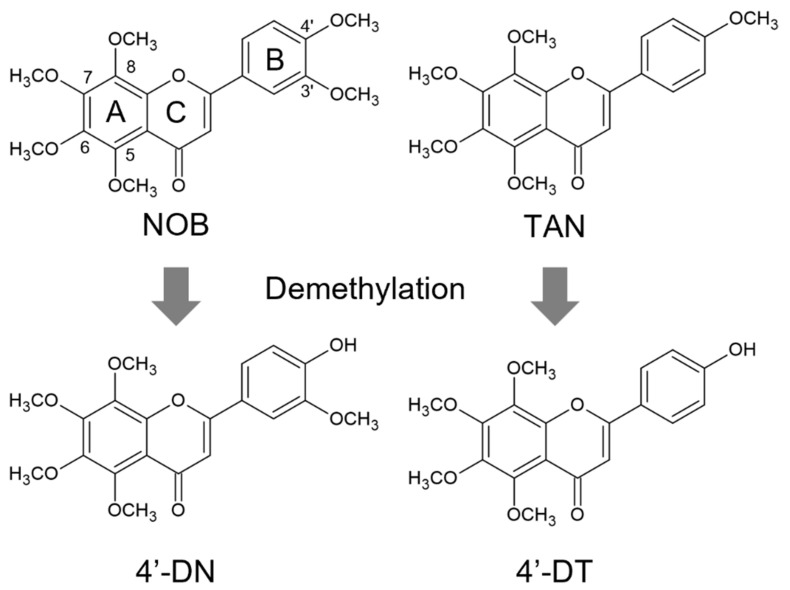
Chemical structures of NOB, TAN, 4′-DN and 4′-DT. Chemical structures of nobiletin (NOB), tangeretin (TAN), 4′-demethylnobiletin (4′-DN) and 4′-demethyltangeretin (4′-DT) were described.

**Figure 2:**
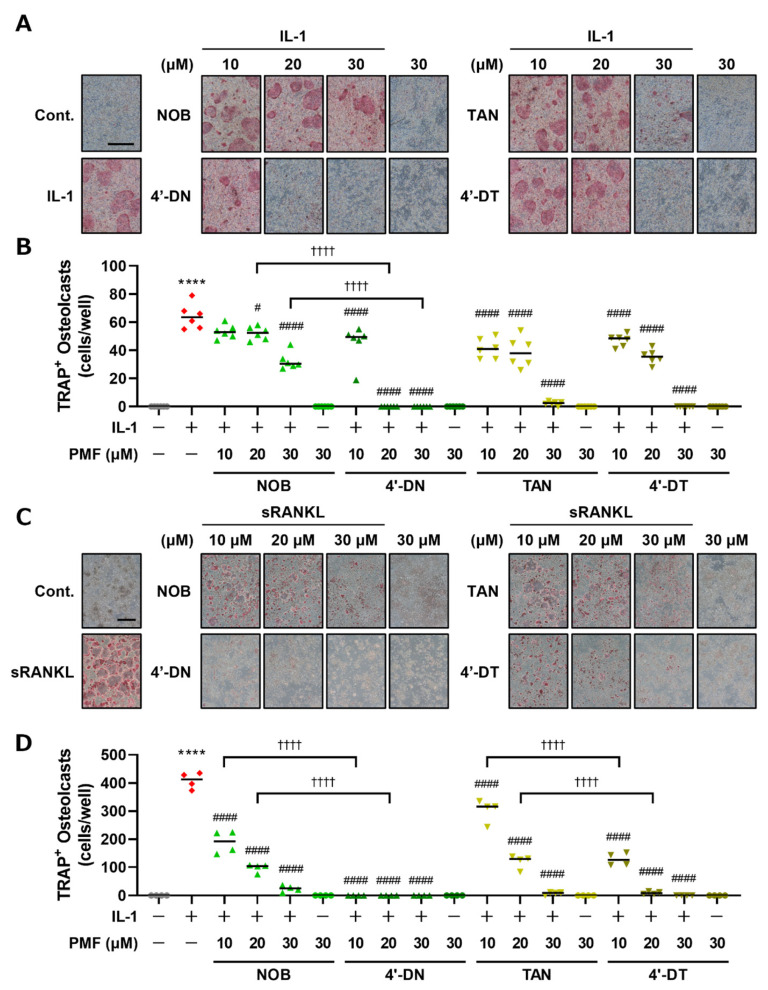
Effects of NOB, TAN, 4′-DN and 4′-DT on osteoclast differentiation. (**A**) POBs and BMCs were cocultured with or without IL-1 (2 ng/mL) and NOB, TAN, 4′-DN or 4′-DT (10, 20 and 30 μM, each) for 7 days. Images show TRAP-stained multinucleated osteoclasts. (**B**) The number of TRAP-stained multinucleated osteoclasts shown in (**A**) was counted. These data are expressed as the median with individual data points (*n* = 6). (**C**) Raw264.7 cells were cultured with or without sRANKL (100 ng/mL) and NOB, TAN, 4′-DN or 4′-DT (10, 20 and 30 μM, each) for 4 days. Images show TRAP-stained multinucleated osteoclasts. (**D**) The number of TRAP-positive multinuclear osteoclasts shown in (**C**) was counted. These data are expressed as the median with individual data points (*n* = 4). The scale bar represents 500 μm. Significant differences between the two groups were indicated as follows: **** *p* < 0.0001 vs. Cont., ^#^ *p* < 0.05 and ^####^ *p* < 0.0001 vs. IL-1 or sRANKL, ^††††^ *p* < 0.0001 by a one-way ANOVA followed by post hoc Tukey’s test.

The chemical structures of NOB, TAN and demethylated 4′-DN and 4′-DT were illustrated using ACD/ChemSketch 2021.2.1 (Advanced Chemical Development, Inc., Toronto, ON, Canada) (Figure 1). We previously demonstrated that 30 μM of NOB and TAN significantly suppressed LPS-induced and sRANKL-induced osteoclast differentiation [9]. To examine the effects of 4′-DN and 4′-DT on osteoclast differentiation, we first utilized two culture systems: coculture of POB and BMC and Raw264.7 culture. In both culture systems, these PMFs dose-dependently suppressed IL-1- or sRANKL-induced osteoclast differentiation (Figure 2). The effect of TAN was greater than that of NOB in cocultures (Figure 2A,B), while that of TAN was lower than that of NOB Raw264.7 cultures (Figure 2C,D). Additionally, demethylated compounds showed a more potent inhibitory effect than NOB and TAN in both culture systems (Figure 2), which is consistent with their previously reported anti-inflammatory properties [13,14].

### 3.2. Mixture of 4′-DN and 4′-DT Suppressed Osteoclast Differentiation in Cocultures of POB and BMC and Raw264.7 Cultures

**Figure 3:**
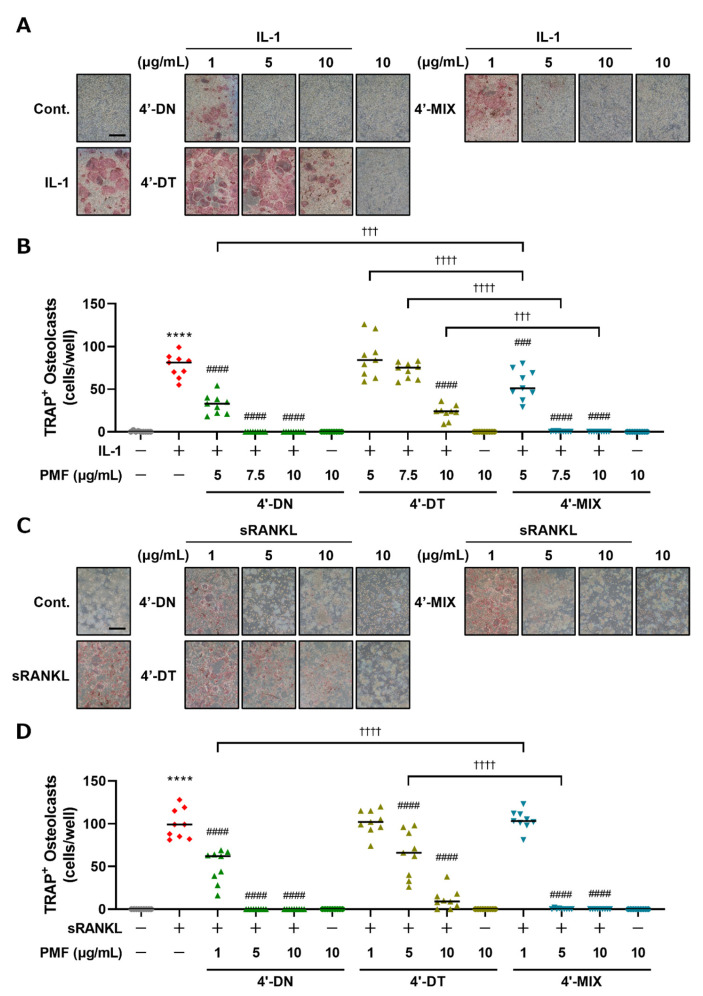
Effects of 4′-DN, 4′-DT and 4′-MIX on osteoclast differentiation. (**A**) POBs and BMCs were cocultured with or without IL-1 (2 ng/mL) and 4′-NOB, 4′-TAN and 4′-MIX (5, 7.5 and 10 μg/mL, each) for 7 days. Images show TRAP-stained multinucleated osteoclasts. (**B**) The number of TRAP-stained multinucleated osteoclasts shown in (**A**) was counted. These data are expressed as the median with individual data points (*n* = 9). (**C**) Raw264.7 cells were cultured with or without sRANKL (100 ng/mL) and 4′-NOB, 4′-TAN and 4′-MIX (1, 5 and 10 μg/mL, each) for4 days. Images show TRAP-stained multinucleated osteoclasts. (**D**) The number of TRAP-positive multinuclear osteoclasts shown in (**C**) was counted. These data are expressed as the median with individual data points (*n* = 9). The scale bar represents 500 μm. Significant differences between the two groups were indicated as follows: **** *p* < 0.0001 vs. Cont., ^###^ *p* < 0.001 and ^####^ *p* < 0.0001 vs. IL-1 or sRANKL, ^†††^ *p* < 0.001, ^††††^ *p* < 0.0001 by a one-way ANOVA followed by post hoc Tukey’s test.

To examine the effects of 4′-MIX, a mixture of 4′-DN and 4′-DT (mixture ratio 1.0:0.6), on osteoclast differentiation, we next compared the effects of 4′-DN, 4′-DT and 4′-MIX on osteoclast differentiation in cocultures of POB and BMC and Raw264.7 cultures. For further in vivo experiments, we purified a large amount of 4′-MIX from citrus peel. In this report, a treatment concentration of 1 μg/mL 4′-MIX is consistent with 4′-DN + 4′-DT:1.6 μM + 1.0 μM treatment. As shown in Figure 3, in both cocultures (Figure 3A,B) and Raw264.7 cultures (Figure 3C,D), 4′-DN, 4′-DT and 4′-MIX dose-dependently suppressed osteoclast differentiation, and the inhibitory effect of 4′-MIX was similar to that of 4′-DN. These data confirm the inhibitory effect of 4′-MIX on osteoclast differentiation.

### 3.3. Mixture of 4′-DN and 4′-DT Inhibits Osteoclast Marker Genes and IκBα Protein Degradation by Adding RANKL

**Figure 4:**
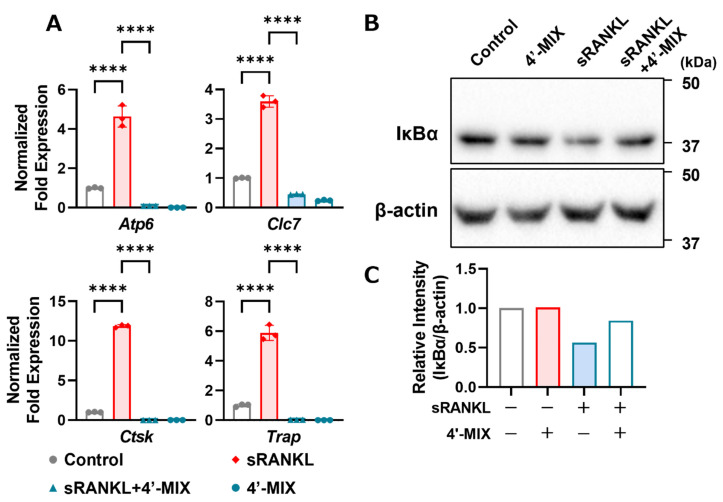
Effects of 4′-MIX on osteoclast differentiation. (**A**) Raw264.7 cells were cultured with or without sRANKL (100 ng/mL) and 4′-MIX (10 μg/mL) for 4 days. The mRNA expression of *Nfatc1* and *Ctsk* was analyzed by RT-qPCR. These data are expressed as the mean ± SD of triplicate measurements from a representative experiment of three independent experiments. The *Actb* gene was used for normalization. Significant differences between two groups were indicated as follows: **** *p* < 0.0001 by a one-way ANOVA followed by post hoc Tukey’s test. (**B**) Raw264.7 cells were cultured with or without sRANKL (100 ng/mL) and 4′-MIX (10 μg/mL) for 15 min. Whole lysates were collected, and the protein expression of IκBα and β-actin was detected by Western blotting. Images of the blots are shown indicated. (**C**) The relative intensity of IκBα protein expression in (**B**) was measured.

To determine the mechanism underlying the demethylation of PMFs, we analyzed the expression of mRNA and proteins. NFATc1 is a master transcription factor for osteoclast differentiation and osteoclast marker genes, such as cathepsin K, TRAP, V-ATPase and chloride channel, are regulated by NF-κB- and NFATc1-dependent transcription activity. As shown in Figure 4A, 4′-MIX downregulated osteoclast marker genes, including cathepsin K (*Ctsk*), TRAP (*Acp5*), V-ATPase (*Atp6*) and chloride channel (*Clc7*), which are involved in resorbing bone matrices. Since our previous study showed that nobiletin suppressed IL-1-induced NF-κB activation during osteoclast differentiation [8], we analyzed the protein expression of IκBα (inhibitor of NF-κB, alpha), an endogenous protein that inhibits NF-κB nuclear translocation. When the NF-κB pathway is activated by several factors, including RANKL, the IκBα protein is rapidly phosphorylated by IKK and degraded via the ubiquitin–proteasome pathway, translocating NF-κB into the nucleus and activating the transcription of its target genes [20]. As shown in Figure 4B, IκBα was degraded by adding RANKL, while its degradation was ameliorated by treatment with 4′-MIX. These data indicated that the inhibitory effects of demethylated PMFs on osteoclast differentiation were mediated by the inhibition of the NF-κB pathway.

### 3.4. In Silico Molecular Docking Simulation of 4′-DN and 4′-DT to ATP Pocket of IKKβ Protein

**Figure 5:**
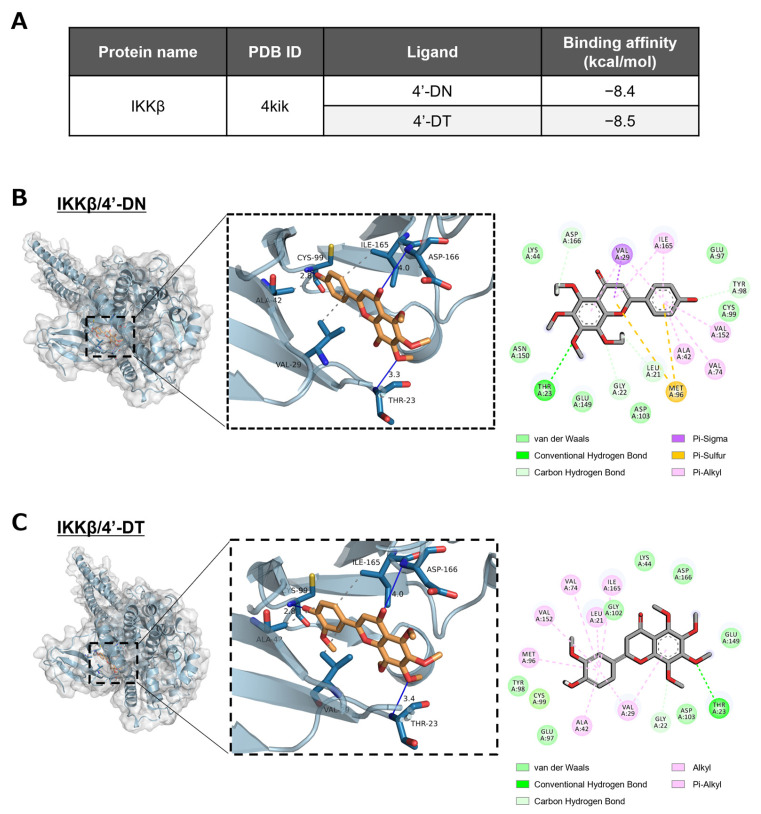
In silico molecular docking simulation of 4′-DN and 4′-DT. (**A**) Semiflexible docking was performed using AutoDock Vina. The binding affinity of 4′-DN and 4′-DT to IKKβ protein was calculated. (**B**,**C**) Three-dimensional docking models of 4′-DN or 4′-DT and IKKβ protein.

The IKK complex, consisting of IKKα, IKKβ and NEMO (IKKγ), is the regulator of the NF-κB signaling pathway, which phosphorylates IκBα and the subsequent degradation of IκBα and nuclear translocation of NF-κB. We examined whether 4′-DN and 4′-DT were able to bind to the ATP-binding pocket of IKKβ protein in an in silico molecular docking simulation. As shown in Figure 5A, the binding affinity of 4′-DN and 4′-DT to the ATP-binding pocket of IKKβ were −8.4 and −8.5 kcal/mol, respectively. These binding affinities were smaller than −7.0, indicating that these compounds can strongly interact with the ATP-binding pocket of IKKβ. Three-dimensional models showed that the 4′-DN was able to form hydrogen-bonding interactions with IKKβ Thr23, Cys99 and Asp166 of IKKβ, with distances of 3.3, 2.8 and 4.0 Å, respectively, and hydrophobic interactions with VAL29, ALA32 and ILE165 of IKKβ (Figure 5B). 4′-DT was capable of forming hydrogen-bonding interactions with IKKβ Thr23, Cys99 and Asp166 of IKKβ with distances of 3.4, 2.8 and 4.0 Å, respectively, and hydrophobic interactions with VAL29, ALA42 and ILE165 of IKKβ (Figure 5C). These data suggested that IKKβ was a possible intracellular target protein of 4′-DN and 4′-DT.

### 3.5. Intraperitoneal Administration of 4′-MIX Inhibits Estrogen Deficiency-Induced Bone Loss in OVX Mice

**Figure 6:**
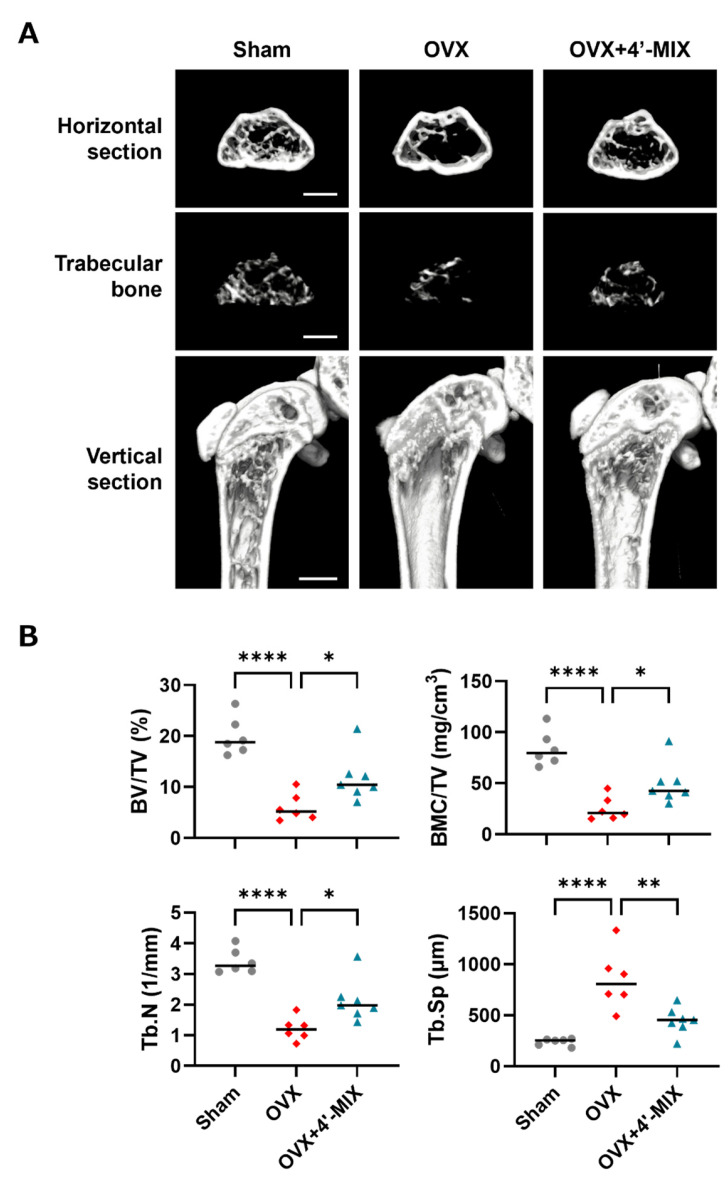
Intraperitoneal administration of 4′-MIX improved bone loss in OVX mice. (**A**) Three-dimensional reconstruction using μCT images of a horizontal section, extracted trabecular bone from the horizontal section and the distal femur from a vertical section. (**B**) The bone microarchitecture parameters, BV/TV (%), BMC/TV (mg/cm^3^), Tb.N (1/mm) and Tb.Sp (μm), were calculated using μCT. These data are expressed as the median with individual data points (*n* = 6–7). The scale bar represents 1 mm. Significant differences between the two groups were indicated as follows: * *p* < 0.05, ** *p* < 0.01 and **** *p* < 0.0001 by a one-way ANOVA followed by Tukey’s post hoc test.

We finally examined the effects of 4′-MIX on bone loss due to estrogen deficiency in OVX mice. 4′-MIX (2 mg/mouse/day) was intraperitoneally administered to OVX mice for 4 weeks. Three-dimensional reconstructed images were obtained at the distal femurs using μCT (Figure 6A). We measured several bone structure parameters in a μCT-based analysis. Bone mineral density is indicated as BMC/TV. BV/TV, Tb.N and Tb.Sp indicate the bone microarchitecture parameters. As demonstrated in Figure 6B, OVX mice showed severe femoral bone loss associated with decreased BMC/TV, BV/TV and Tb.N and increased Tb.Sp; however, the intraperitoneal administration of 4′-MIX significantly improved these parameters in OVX mice.

## 4. Discussion

Various polyphenols and PMFs have been reported to have various beneficial effects (antioxidant effects, anti-inflammation effects and the prevention of metabolic bone diseases). We achieved the demethylation of NOB and TAN using an enzyme manufacturing method, which resulted in 4′-DN and 4′-DT. These demethylated compounds are contained in fermented citrus [13,14,15]. NOB exhibited a lower inhibitory effect than TAN on osteoclast differentiation in cocultures (Figure 2A,B), whereas the effects of NOB were greater than those of TAN in Raw264.7 cultures (Figure 2C,D). These data are consistent with our previous report [9]. The difference in the effect of NOB and TAN may have resulted from the use of different types of cell cultures—cocultures use two types of cells, including bone marrow cells as osteoclast precursor cells and osteoblasts, while Raw264.7 cultures only use osteoclast precursor cells. In this study, 4′-DN and 4′-DT showed stronger inhibitory effects against osteoclast differentiation in comparison to NOB or TAN in cocultures, and in Raw264.7 cultures (Figure 2). 4′-DN also exhibited more potent inhibitory effects in comparison to 4′-DT in both culture systems. Furthermore, 4′-MIX, the mixture of 4′-DN and 4′-DT, showed inhibitory effects on osteoclast differentiation. These inhibitory activities were very similar to those of 4′-DN (Figure 3).

Li et al. reported that among NOB and demethylated compounds, including 3′-demethylnobiletin (3′-DN), 4′-DN and 3′,4′-didemethylnobiletin (3′,4′-DN), both 4′-DN and 3′,4′-DN possessed the most potent anti-inflammatory activities [13]. Wang et al. reported that the antioxidative effects of 5-demethylnobiletin (5DN) and 5-demehyltangeretin (5DT) were stronger than those of NOB and TAN [21].

One of the explanations for the different activities among 4′-DN and 4′-DT is the inhibitory target and its inhibitory activities. Our present study clearly showed that these demethylated compounds induced the degradation of IκBα in Raw264.7 cells (Figure 4B). The degradation of IκBα led to the inhibition of the NF-κB signaling pathway and the downregulation of osteoclast differentiation. Our previous reports also indicated that natural compounds, including nobiletin, delphinidin and β-cryptoxanthin, directly bound to IKKβ and inhibited the kinase activity of IKKβ [8,22,23].

To further confirm the molecular target of IKKβ for 4′-DN and 4′-DT, we demonstrated an in silico molecular docking simulation using IKKβ. The results showed that 4′-DN and 4′-DT are capable of forming hydrogen-bonding interactions with IKKβ Thr23 (3.3 Å and 3.4 Å, respectively), Cys99 (2.8 Å each) and Asp166 (4.0 Å each) of IKKβ (Figure 5). 4′-DN possibly forms hydrophobic interactions with VAL29, ALA32 and ILE165 of IKKβ, and 4′-DT might be able to form hydrophobic interactions with VAL29, ALA42 and ILE165 of IKKβ (Figure 5). The difference in the inhibitory activities of 4′-DN and 4′-DT against osteoclast differentiation is possibly explained by the difference in accessibilities and binding affinity of 4′-DN and 4′-DT to the ATP-binding pocket of IKKβ. Further studies are needed to confirm whether the location and conversion of methoxy moiety into the hydroxyl group at the 4′-position on the B-ring, and 5-position on the A-ring in 4′-DN and/or 4′-DT enhance their biological activities.

We finally examined the in vivo effect of 4′-DN and 4′-DT. Treatment with 4′-MIX attenuated estrogen withdrawal-induced bone loss (Figure 6). OVX mice showed severe femoral bone loss associated with bone-decreased parameters and 4′-MIX intraperitoneal administration significantly recovered bone mass in OVX mice. Our previous report also indicated that the intraperitoneal administration of NOB showed anti-osteoporotic effects in OVX mice [8]. The local injection of NOB or TAN suppressed LPS-induced inflammatory bone resorption in a mouse model of periodontitis [9]. We also reported that the oral administration of a PMF mixture (5 mg/mouse/day), which consisted of NOB (35.7%), TAN (11.0%), heptamethoxyflavone (2.4%) and tetramethoxyflavone (38.8%), suppressed bone loss in OVX mice [11]. In other reports, Murakami et al. showed that treatment with NOB suppressed bone loss in OVX mice [24] and Wang et al. demonstrated that the delivery of NOB-containing micelles prevented ovariectomy-induced bone loss in mice [25]. In the present study, we found again that the intraperitoneal administration of a mixture of 4′-MIX ameliorated estrogen deficiency-induced bone loss in OVX mice. Further pathways of administration are needed to examine the effects of 4′-MIX in OVX mice.

## 5. Conclusions

In conclusion, the present study demonstrated that 4′-DN and 4′-DT inhibit osteoclast differentiation in cultures and bone loss in OVX mice. We found that these compounds bound directly to IKK and blocked the NF-κB pathway via the direct inhibition of IKK activity, leading to the suppression of osteoclast differentiation. These demethylated compounds are novel candidates for maintaining bone health in the elderly population who are at risk for the development of metabolic diseases such as osteoporosis.

## Data Availability

The datasets used and/or analyzed during the current study are available from the corresponding author upon reasonable request.
